# Resurrection ecology: An approach for understanding eco‐evolutionary plant‐microbe interactions under global change

**DOI:** 10.1002/ajb2.70218

**Published:** 2026-06-09

**Authors:** Candice Y. Lumibao

**Affiliations:** ^1^ Department of Life Science Texas A&M University–Corpus Christi Corpus Christi 78412 Texas USA

**Keywords:** ancestral microbes, ancestral plant, endophytes, marsh seed banks, multi‐generation plant‐microbe feedback, plant microbiomes, rhizosphere, *Schoenoplectus americanus*

Plant‐microbe interactions span a continuum of relationships—from antagonistic/negative to mutualistic/positive—that occur both below and above ground: in the rhizosphere (within ~2 mm of plant roots), the endosphere (inside plant tissues), and the phyllosphere (surfaces of aerial organs). These include pairwise partnerships, whereby individual plants associate with a specific microbe (e.g., mycorrhiza), as well as diffuse interactions between plants and diverse communities of microbes. These interactions are subject to eco‐evolutionary processes—the feedback between ecological and evolutionary change (Hendry, 2020)—that can be influenced by global change (Angulo et al., 2022). For example, microbes can influence both the ecological (e.g., growth; Burghardt, 2020; Lumibao et al., 2022) and the evolutionary responses (i.e., through altered strength of selection on plant traits) of plants to global change (Friesen et al., 2011). In turn, plants can shape the microbe's ecological (e.g., population changes; Lumibao et al., 2025) and evolutionary responses, initiating eco‐evolutionary interactions. However, temporal constraints associated with long generation times of plants present a challenge in capturing evolutionary change within an ecological timescale that can impact the outcomes of ongoing ecological interactions. While recent evidence suggests that plants are capable of rapid, contemporary evolution (i.e., within one to ten generations; Christie et al., 2023; Pennington et al., 2025 [preprint]), longer timescales may be needed to capture eco‐evolutionary processes underlying plant‐microbe interactions. For example, perennial plants may require centuries or millennia to manifest evolutionary responses mediated by microbes. “Resurrection ecology,” or the revival of long‐dormant propagules, offers a powerful approach for understanding eco‐evolutionary plant‐microbe interactions (e.g., trajectory, nature, and feedback mechanism).

## RESURRECTION ECOLOGY IN ECO‐EVOLUTIONARY PLANT‐MICROBE INTERACTIONS

The revival of dormant propagules—such as daphnia eggs or plant seeds, stored in their natural environment (Frisch et al., 2020; Blum et al., 2021), in archival seeds banks, or in herbarium collections (Christie et al., 2023)—has long been used in eco‐evolutionary studies to track organismal or population responses to global change (Franks et al., 2018; Summers et al., 2018; Vahsen et al., 2023). These dormant propagules represent spatiotemporal integrators of genomic‐, phenotypic‐, species‐, and community‐level variation, revealing important evolutionary insights, including adaptations to current environmental conditions (Karitter et al., 2024), genotypic versus plastic responses, and the phenotypic evolution of species or populations (Frisch et al., 2020; Vahsen et al., 2023). There is good reason to think that resurrection approaches can also yield powerful insights into the eco‐evolution of plant‐microbe interactions under global change (i.e., climate‐related shifts in environmental conditions such as increasing salinity, drought, and temperature).

**Figure 1:**
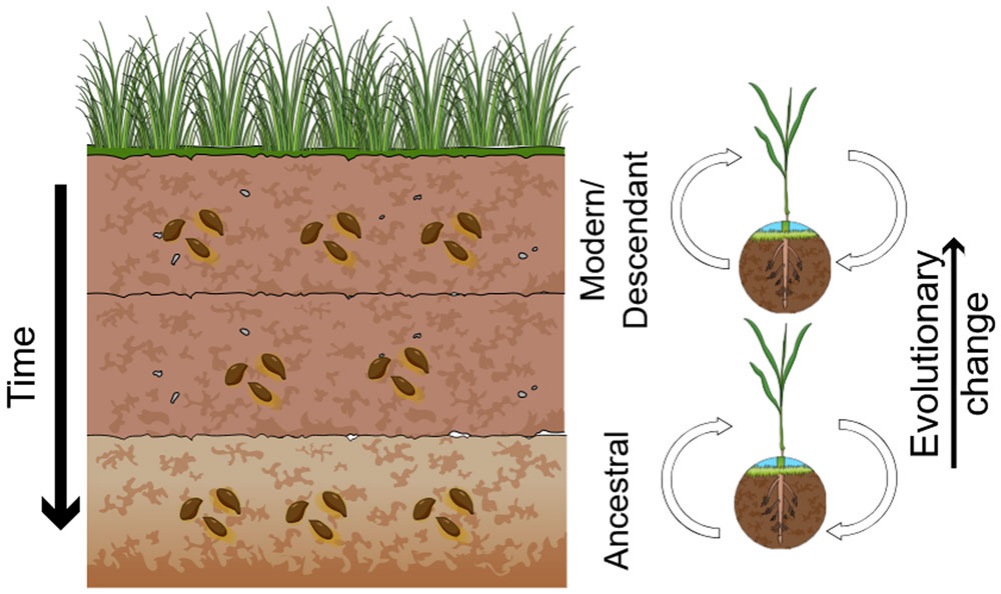
Eco‐evolutionary dynamics of plant‐microbe interactions can shift across time. “Resurrecting” and germinating seeds preserved in natural environments (e.g., marshes) and comparing the plants with modern lineages could shed light into how evolution of traits can impact ongoing microbial interactions (and vice versa) within ecological timescales.

Resurrection studies comparing ancestral and descendant plant genetic lineages provide an avenue for tracking plant‐microbe associations over time and predicting how they might further shift or evolve with global change. For example, genetic changes underlying differences in plant traits between ancestral and descendant lineages (i.e., evolution of plant traits) might give rise to different degrees and direction of microbial associations (Figure 1). If the evolution of plant traits such as root exudates or biomass production amplifies the growth of beneficial suites of microbes (Porter and Sachs, 2020), the microbial community composition and functioning may change over time (Lumibao et al., 2022). Changes in plant traits may also increase selection of microbial traits, such as production of the plant growth‐promoting hormone gibberellin. Such variation across plant generations may become even more pronounced if the strength of trait selection imposed by environmental change differs between ancestral and modern plants, leading to differential shifts in microbial recruitment and, hence, in the direction and strength of associations over time. For example, a common‐garden experiment with the marsh sedge *Schoenoplectus americanus* showed that modern plants exhibited reduced root biomass allocation and shift in root depth distribution compared to ancestral plants, likely in response to selection imposed by nitrogen enrichment in recent years (Vahsen et al., 2023). Experiments involving exposure of these ancestral and descendant lineages to a common environmental condition coupled with microbial inoculation will yield information on how trait evolution due to global change can lead to changes in microbial recruitment across plant generations.

Differences between ancestral and descendant/modern plants may also arise from variation in the magnitude of microbe‐driven selection of plant traits that counteract negative effects of global change, which can then feed back into microbial associations. Shifts in microbial associations may further lead to varying magnitudes of microbial mediation of plant responses to environmental change between ancestral and descendant lineages. For example, the microbial mediation of the salinity stress response of *S. americanus* differed in strength between ancestral and modern/descendant plant lineages, depending on the plant provenance and plant traits considered (Lumibao et al., 2022). Ancestral plants tend to grow taller and produce fewer shoots in the presence of a native soil microbiome compared to modern plants, but only under high salinity stress. In turn, ancestral and modern plant lineages recruit distinct rhizosphere microbial communities that reflect genotypic trait differences when inoculated with “contemporary” soil microbiomes under abiotic stress, with ancestral genotypes more likely to recruit distinct fungal guilds (e.g., decomposers and endosymbionts) than modern plants under high abiotic stress (Y. Liu, M. J. Blum, and C. Y. Lumibao, unpublished data).

While resurrecting seeds is the common approach, reviving and cultivating dormant plant‐associated microbes (e.g., isolating individual ancestral seed endophytes) or retrieving ancestral soil microbiomes (community‐level) will deepen our understanding of the evolution of plant‐microbe associations. Presumably, these ancestral microbes/microbiomes were in stable partnership with ancestral plants. They can thus serve as a baseline for assessing how these interactions have evolved over time, as well as the magnitude of shifts in interactions that may occur in response to future global change. Insights can be gained into the (mal)adaptation of plants to environmental shifts driven by the strength of their microbial associations (Barnes and Tringe, 2022), as well as the coadaptation and coevolution between plants and microbes through time. Reciprocal microbial inoculation experiments, in which ancestral and modern plants are inoculated with their respective microbes/microbiomes (i.e., “home”) and vice versa (i.e., “away”) and subsequently exposed to common environments, will allow for quantifying reciprocal benefits (e.g., fitness) and ascertaining the degree of coadaptation/coevolution between plants and microbes. They can also give insights into how the strength of reciprocity changes as the associations evolve in response to global change. Further information can also be gained on the rate and direction of shifts in these associations, while accounting for changes in the genotypic and phenotypic compositions of the plants and associated microbes as they respond to global change. Comparison between ancestral‐ancestral and modern‐modern plant‐microbe interactions can also be used to explore multigenerational eco‐evolutionary feedback, whereby microbes “conditioned” by parent plants act as a selective agent on offspring trait variation (Panke‐Buisse et al., 2014). This will involve, first, growing ancestral and modern parent plants in a similar soil environment and/or other environmental conditions with their respective soil microbes. Generating their progenies and exposing them to the same soil‐microbe environment conditioned by their parents will offer insights about the strength of the ancestral versus modern parent soil/microbial legacy for (and its feedback into) their offspring (e.g., selection of offspring traits).

**Figure 2:**
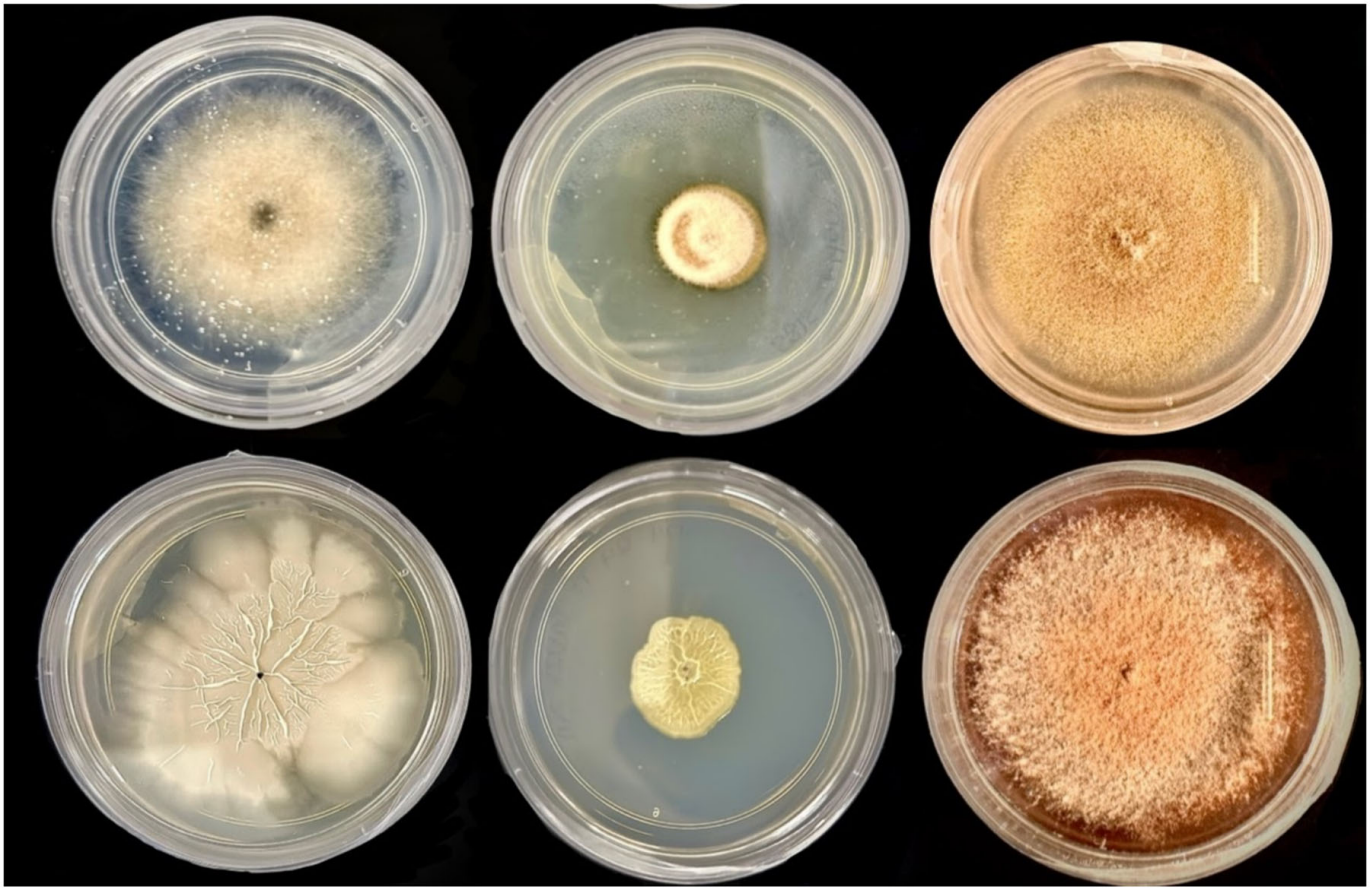
Collection of endophytes isolated from ancestral *Schoenoplectus americanus* seeds retrieved from Chesapeake Bay.

Notably, reviving dormant individual plant‐associated microbes or retrieving “ancestral” microbiomes (especially those in rhizosphere soil) from their natural environment may be challenging due to elapsed time or sediment accretion and movement. A pilot study, however, showed promising evidence that “ancestral” endophytes can be isolated and cultivated from approximately 100‐year‐old *S. americanus* seeds, which can be used for inoculation experiments (T. Storr and C. Y. Lumibao, unpublished data; Figure 2). In cases where ancestral microbes cannot be retrieved, studies that utilize contemporary microbiomes for inoculation experiments of ancestral and modern plants would still be valuable. They can still, for example, provide insight into how microbes in general influence expression of plant traits in ancestral and descendant lineages (and the eco‐evolutionary feedback) under global change.

## CONCLUSIONS

Plants and microbes interact in ways that influence both their ecological and evolutionary responses to changing environments. Global changes are profoundly altering the strength and trajectories of plant‐microbe interactions. An eco‐evolutionary framework is critical in determining and predicting the resilience of both plants and microbes to future environmental shifts. Resurrection ecology facilitates better elucidation of the eco‐evolutionary processes underlying these interactions by providing a baseline for evaluating the direction, strength, and rate of change in these associations as global change proceeds.

## Data Availability

Data sharing is not applicable to this article as no new data were created or analyzed in this study.
